# Molecular forensics in avian conservation: a DNA-based approach for identifying mammalian predators of ground-nesting birds and eggs

**DOI:** 10.1186/s13104-015-1797-1

**Published:** 2016-01-07

**Authors:** Matthew W. Hopken, Elizabeth K. Orning, Julie K. Young, Antoinette J. Piaggio

**Affiliations:** United States Department of Agriculture, Wildlife Services, National Wildlife Research Center, Fort Collins, CO USA; Department of Wildland Resources, Utah State University, Logan, UT USA; Oregon Cooperative Fish and Wildlife Research Unit, Department of Fisheries and Wildlife, Oregon State University, Corvallis, OR USA; United States Department of Agriculture, Wildlife Services, National Wildlife Research Center, Logan, UT USA

**Keywords:** Bighorn Basin, *Canis latrans*, *Centrocercus urophasianus*, Greater sage-grouse, Nest predation, Non-invasive sampling, Wyoming

## Abstract

**Background:**

The greater sage-grouse (*Centrocercus urophasianus*) is a ground-nesting bird from the Northern Rocky Mountains and a species at risk of extinction in in multiple U.S. states and Canada. Herein we report results from a proof of concept that mitochondrial and nuclear DNAs from mammalian predator saliva could be non-invasively collected from depredated greater sage-grouse eggshells and carcasses and used for predator species identification. Molecular forensic approaches have been applied to identify predators from depredated remains as one strategy to better understand predator–prey dynamics and guide management strategies. This can aid conservation efforts by correctly identifying predators most likely to impact threatened and endangered species. DNA isolated from non-invasive samples around nesting sites (e.g. fecal or hair samples) is one method that can increase the success and accuracy of predator species identification when compared to relying on nest remains alone.

**Results:**

Predator saliva DNA was collected from depredated eggshells and carcasses using swabs. We sequenced two partial fragments of two mitochondrial genes and obtained microsatellite genotypes using canid specific primers for species and individual identification, respectively. Using this multilocus approach we were able to identify predators, at least down to family, from 11 out of 14 nests (79 %) and three out of seven carcasses (47 %). Predators detected most frequently were canids (86 %), while other taxa included rodents, a striped skunk, and cattle. We attempted to match the genotypes of individual coyotes obtained from eggshells and carcasses with those obtained from fecal samples and coyotes collected in the areas, but no genotype matches were found.

**Conclusion:**

Predation is a main cause of nest failure in ground-nesting birds and can impact reproduction and recruitment. To inform predator management for ground-nesting bird conservation, accurate identification of predator species is necessary. Considering predation can have a high impact on recruitment, predation events are very difficult to observe, and predator species are difficult to identify visually from nest remains, molecular approaches that reduce the need to observe or handle animals offer an additional tool to better understand predator–prey dynamics at nesting sites.

**Electronic supplementary material:**

The online version of this article (doi:10.1186/s13104-015-1797-1) contains supplementary material, which is available to authorized users.

## Background

Non-invasive genetic sampling has become a fundamental tool in wildlife management and conservation. The ability to collect genetic information without handling or directly observing animals has provided opportunities to explore ecological processes that are often difficult to measure [1, 2]. For example, wildlife forensic approaches utilizing non-invasive genetic samples have been applied to management questions surrounding depredation at both wild and domestic animal kill sites [3–5]. A multitude of questions have been addressed with non-invasive genetic samples from depredation sites such as which wildlife or feral species kill livestock, which predators are most likely to focus on game species, do invasive predators kill native wildlife, and which predators are likely to feed upon endangered or threatened species [3, 5, 6]. Answering the latter question is very important for development of predator management plans to protect endangered species as response to predation is often species-specific. Accurate species identification and quantification of various predator species improves management efficiency and reduces non-target impacts by focusing efforts on known predator species that most heavily impact endangered populations.

Nest predators are often challenging to identify due to the lack of species-specific signs left at nests, and because depredation events are seldom observed directly [7, 8]. Improvements to nest predator species identification have been made through the use of technology, such as continuous video monitoring and infrared trail cameras, but these methods can be labor intensive, time-consuming, and may alter predator behavior [9–11]. Further, the lack of evidence left at nests limits the use of some types of non-invasive DNA samples typically employed to identify predator species, such as hair and feces around kill sites [4, 5]. The goal of this study was to test the concept that non-invasive genetic sampling techniques can assist identification of mammalian predators of greater sage-grouse (*Centrocercus urophasianus*; hereafter sage-grouse) nests and carcasses through isolation of mammalian predator DNA from depredated remains. Sage-grouse are considered species of concern by the state of Wyoming and a species at risk by the Canadian government. The species is a ground-nesting bird which leaves the roosting hens and eggs vulnerable to a broad range of terrestrial predators [10]. Previous work has demonstrated that sage-grouse chick DNA can be isolated from eggshells [12] and that avian predator DNA can be isolated from sea bird eggshells and carcasses [13]. Thus, we expanded on the single locus method employed by Steffens et al. [13] by using both nuclear and mitochondrial DNAs for species and individual identification and to test the applicability of these methods for detection of mammalian predators from sage-grouse eggshells. We expect to further substantiate the use of molecular forensics for avian conservation and management as it allows the identification of depredating species and can provide managers with information on cause-specific mortality during important reproductive time periods.

## Methods

### Study species

Throughout the western United States and Canada populations of sage-grouse have declined to the extent that the species is now listed as endangered under Canada’s Species at Risk Act and proposed for listing under United States’ Endangered Species Act [14–16]. Sage-grouse currently occupy a significant portion of the sagebrush steppe throughout much of the state of Wyoming. The three factors with the greatest influence on sage-grouse population growth rates are hen survival, nest success, and recruitment; each can be heavily impacted by predation [17–19].

### Sample collection

This study was conducted in the northwest portion of Bighorn Basin, Wyoming. We conducted the study at three lek complexes: Oregon Basin (44° 22.45 N, 108° 48.17 W), 15 Mile (44° 10.89 N, 108°44.38 W), and Polecat Bench (44° 57.00 N, 108° 45.54 W). Sage-grouse nests were monitored via telemetry of hens and infrared trail cameras (Bushnell Outdoor Products, Overland Park, Kansas, USA) placed at a nest’s entrance or exit and left in place until nests hatched or failed [20]. We monitored hens to confirm survival and their location on nests. When a nest failed, we collected egg remains between 1 and 4 days post-predation. When hen mortality was detected we collected carcasses the day of detection with a mean detection of 3.5 days after predation (range: 0–8 days). All samples were placed into re-sealable plastic bags, stored at −20 °C, packed tightly with soft, absorbent material, and shipped on ice the same day of collection. Once the samples arrived in the laboratory they were stored at −80 °C for a maximum of 5 days until DNA extraction.

We used cotton swabs (Fisher Scientific, USA) wetted with a few drops (1–3) of ultra-pure water to swab carcasses and eggshells. For each individual egg, we used one swab on the inside and one swab on the outside of the egg. We used a single swab for each carcass and targeted regions on feathers that appeared matted from saliva and or had bite marks. The swabs were air dried and the tips were removed with sterile razor blades and extracted using the QIAmp DNA Micro Kit (Qiagen, Valencia, CA, USA) following the “Isolation of Genomic DNA from Swabs protocol.” We eluted with 50 µL of Buffer AE and ran the buffer through the column two times with 5 min incubation at room temperature each time. All DNA extractions were completed in a room and biosafety cabinet with reagents and laboratory supplies dedicated to non-invasive extractions and included extraction blanks (reagents only) for each extraction to monitor contamination.

### PCR, mitochondrial DNA sequencing, and nuclear DNA genotyping

We initially employed the general mammalian primers MVZ04 and MVZ05 to amplify approximately 400 bp of the mitochondrial cytochrome-b gene [21]. However, in some cases this gene fragment failed to amplify and lacked variation to differentiate *Canis* species. Thus for those samples we amplified approximately 350 bp of the mitochondrial control region using primers L15926 and H16340 [22]. Cytochrome-b (cyt-b) was amplified in a 25 µL reaction using Amplitaq Gold 10× buffer II (Life Technologies, USA), 1.5 mM MgCl_2_, 0.25 mM of each dNTP, 0.4 µM of each primer, 2 units of Amplitaq Gold polymerase, and 2 µL of DNA extract. The control region was amplified in an identical reaction solution to cyt-b but with 1 µM of each primer. Both genes were amplified on Eppendorf Mastercycler EP with the following program: initial denaturation at 95 °C, 40 cycles of 94 °C for 30 s, 48 °C for 45 s (cyt-b) or 46 °C for 30 s (control region), and extension at 72 °C for 1 min. We included a final extension at 72 °C for 7 min. We subjected each extraction blank to PCR and included negative controls for each reaction to monitor contamination. When contamination was detected, we reran the PCR once with fresh aliquots of reagents to determine if contamination occurred in the PCR, extraction, or field collection. Products from successful PCRs where purified using ExoSAP (Affymetrix, USA) and sequencing reactions for both directions were performed using BigDye v 3.0 (Life Technologies, USA). We used ¼ the manufacturers recommended amount of BigDye terminator RR-100 in 10 µL reaction containing 5× buffer, 1 µM of either forward or reverse primer, and 1 µL of PCR product. Sequences were run on an Applied Biosystems 3130xl Genetic Analyzer (Life Technologies, USA). Sequences were aligned and edited with sequencher v4 (Gene Codes, USA). Final species identification was completed through evaluation against GenBank using BLAST. Identity scores greater than 95 % were used as final criteria for species identification [23].

When we determined that the individual predator was a canid with mtDNA sequences but still could not obtain species identification due to haplotype similarity, we employed eight microsatellite loci (Set A) to increase the resolution of species identification (Table 1; [24, 25]. If the predator species was determined to be coyote (*Canis latrans*) we then amplified a second set of 10 microsatellite loci [Set B; 25] to identify individual animals and compare genotypes obtained from nests and carcasses and to those collected from feces and coyotes removed from sage-grouse leks during the course of this study [20]. The reason we employed two sets of microsatellite loci is that our laboratory already had developed a canid identification database based on set A but Orning [20] used set B for individual genotypes. Thus, to determine both species and individual identification we needed to use both sets. All microsatellite reverse primers were PIG-tailed to facilitate accurate genotyping [26]. We performed PCR for Set A in three multiplexes using 10 µL reactions that contained Amplitaq Gold 10× buffer II, 3 mM MgCl_2_, 0.25 mM of each dNTP, 2 units of Amplitaq Gold polymerase, 2 µG of bovine serum albumin, and 2 µL of DNA extract. Primer concentrations are provided in Table 1. The thermocycler program for all three multiplexes in Set A was 95 °C for 15 min, 40 cycles of 94 °C for 15 s, 58 °C for 15 s, and extension at 72 °C for 30 s. We included a final extension at 60 °C for 30 min. All three multiplexes for Set B had similar 10 µL mixes that contained Amplitaq Gold 10× buffer II, 2.5 mM (Multiplexes A and B) or 2.0 mM (Multiplex C) of MgCl_2_, 0.25 mM of each dNTP, 1 unit of Amplitaq Gold polymerase, 2 µG of bovine serum albumin, and 2 µL of DNA extract. Primer concentrations are provided in Table 1. The thermocycler program for Set B was 95 °C for 10 min, 52 cycles of 94 °C for 30 s, annealing temperature (Table 1) for 30 s, and extension at 72 °C for 45 s and a final extension at 72 °C for 7 min. Each PCR was run three times to estimate allelic dropout or false alleles [27]. We only scored an allele if it was present in at least two of the PCR replicates [28]. False alleles and allelic dropout were estimated with gimlet v1.3.3 with the consensus genotype threshold set at two [29].

**Table 1:** Microsatellite multiplexes used to genotype predator DNA from greater sage-grouse eggs and carcasses

	Annealing temp	Concentration (µM)
Set A		
Multiplex A	58	
172		0.25
200		0.75
204		0.25
Multiplex B	58	
123		0.3
225		0.3
250		0.3
Multiplex C	58	
109		0.6
344		0.35
Set B		
Multiplex A	51	
FH2001		0.4
FH2096		0.35
FH2137		0.25
CX140		0.3
Multiplex B	50	
FH2054		0.3
FH2010		0.3
FH2159		0.5
Multiplex C	59	
CX2235		0.5
FH2100		1.1
FH2062		0.35

Loci and multiplexes are listed in the first column. Also included are annealing temperatures for each multiplex and primer concentrations

Species identification of canid genotypes (Set A) was determined using the Bayesian clustering algorithm in structure v2.3.4 [30, 31]. We compiled a database that included dogs (*Canis familiaris*; n = 34), coyotes (n = 69), western North American wolves (*C. lupus*; n = 50), and wolves from Minnesota and Wisconsion (*C. lycaon* and *C. lupus*; n = 109) (A. J. Piaggio; unpublished data). The genotypes from the eggs were compared to this database using structure with the admixture and allele frequencies correlated models, burn-in of 70,000, and MCMC length of 700,000. The analysis was run with *k* set at six and replicated five times.

Orning [20] estimated the number of coyotes around sage-grouse leks and genotyped lethally removed coyotes and fecal samples collected along transects. We attempted to obtain genotypes from eggs and carcasses to identify individual coyotes from the area using microsatellite Set B. We used the genotype match function in genalex v6.5 [32] to compare canid genotypes from eggs and carcasses to the coyote genotypes collected by Orning [20].

## Results

A total of 14 depredated nests were discovered and we recovered an average depredated egg count of 4.2 (range: 1–8; Table 2) per nest. Using the genetic methods described herein, we successfully amplified mammalian mtDNA from 11 of 14 nests (78.5 %; Table 2).The average number of eggs per nest from which we obtained predator identification was 2.7 (range 0–8; Table 2). We also opportunistically collected 7 hen carcasses. We were successful at obtaining species identification from three out of seven carcasses (43 %). The most common predator taxa identified (9 out of 14 nests; 3 out of 7 carcasses; total = 86 %), were canids. From the other 14 % we identified striped skunk (*Mephitis mephitis*), deer mouse (*Peromyscus maniculatus*), and cattle (*Bos taurus*). The approach of using two mtDNA genes increased our ability to identify predators (Table 2). When we used only the cyt-b gene, we were able to identify nest predators in 57 % of cases. However, when we subsequently employed the control region for those samples that failed to amplify with cyt-b primers, our success rate went up to 79 %. We did encounter problems with human contamination that seemed to primarily source from field collection but there were two instances where it originated in the lab.

**Table 2:** Results from molecular identification of greater sage-grouse nest and carcass predators using both mitochondrial and microsatellite DNAs

Nest (N) or carcass (C) ID	# Eggs	# Successful sequences		# msat genotypes		Sequence ID from BLAST	STRUCTURE assignment	Molecular ID	Field cues	Camera traps
		CB	CR	Set A	Set B					
N1	7	2				99 % cow		Cow	Bird or Bobcat	Indeterminate
N2	3	1				98 % coyote		Coyote	Cow	Cow
N3	4	2				99 % coyote		Coyote	Coyote	Coyote
N4	7	1	2	4	1	99 % coyote	97 % coyote	Coyote	Coyote	Coyote
N5	7	1	3	1		99 % coyote	97 % coyote	Coyote	Coyote	Coyote
N6	1							Indeterminate	Indeterminate	Raven
N7	3	3		1	1	99 % coyote/99 % wolf (WI)	38 % coyote/37 % wolf (WY)/17 % wolf (WI)	Wild canid	Raven or coyote	Indeterminate
N8	8	8	1	1		99 % coyote/99 % wolf (WI)	98 % coyote	Coyote	Magpie or coyote	Coyote
N9	1		1			99 % dog		Dog	Raven or snake	Indeterminate
N10	1	1				97 % striped skunk		Striped skunk	Indeterminate	Striped skunk or badger
N11	8		3	1		100 % dog/99 % deer mouse	92 % coyote	Coyote/dog/deer mouse	Indeterminate	Indeterminate
N12	1		1			dog		Dog	Indeterminate	Weasel
N13	3							Indeterminate	Indeterminate	Indeterminate
N14	6							Indeterminate	Indeterminate	Indeterminate
							Percent nests with molecular ID: 78.6 %			
C1	NA							Indeterminate	Indeterminate	NA
C2	NA	Y		1	1	100 % coyote/100 % wolf (WI)	96 % coyote	Coyote	Coyote	NA
C3	NA	Y		1	1	98 % coyote/98 % wolf (WI)	76 % coyote/20 % dog	Canid	Indeterminate	NA
C4	NA							Indeterminate	Indeterminate	NA
C5	NA	Y			1	99 % coyote/99 % wolf (WI)		Wild canid	Indeterminate	NA
C6	NA							Indeterminate	Indeterminate	NA
C7	NA							Indeterminate	Indeterminate	NA
							Percent carcasses with molecular ID: 42.9 %			

Listed in the table are ID for each depredated carcass or nest, the total number of eggs collected from each nest (# eggs), the number of eggs with successful predator ID using DNA sequences (# successful sequences; *CB* cytochrome b, *CR* control region), # of microsatellite genotypes used for species idenitifcation (# msat genotypes for set A and set B), species identification using genotypes and assigned in STRUCTURE (STRUCTURE assignment), identification of predator based on sequences, microsatellites, or both (molecular ID), predator identification in the field from nest remains (Field cue), and predator identification based on trails cameras placed on nest. The percent match was generated from BLAST. *WY* Wyoming and *WI* Wisconsin

We successfully obtained complete genotypes with microsatellite Set A from eight eggs obtained from five nests (Additional file 1). The set A allelic dropout rate across loci was 0.095 and the false allele rate across loci was 0.089. These genotypes allowed us to distinguish which canid species were the nest predators where we lacked resolution with mtDNA (Table 2). Two of the five nest genotypes (N4 and N5) confirmed mtDNA results and identified the predator as coyote. Microsatellite set A also helped us refine the species identification for one nest (N8), which was a coyote, where the mtDNA could not distinguish between wolf or coyote. Neither the microsatellites nor the mtDNA could resolve whether the nest predator for N7 was a coyote or wolf, thus we classified it as a wild canid. The mtDNA extracted from two eggs from a single nest (N11) identified dog as the predator, although the sequence recovered from one egg had only an 88 % identity match while the other was 100 % (Table 2). This was the same nest from where we also extracted and amplified deer mouse mtDNA from one egg. The single canid genotype we obtained from N11 further identified that a coyote was also in contact with this nest. Overall, we identified coyotes from six nests, dogs from three nests, and one we could not distinguish whether wolf or coyote, thus we called it a wild canid (likely coyote as wolves are not common in this area [33]). Predators of three of the hen carcasses were coyotes (n = 2) and a wild canid (n = 1) as identified with both mtDNA and microsatellites (Table 2).

We obtained six genotypes (Set B) from nests and carcasses that contained coyote DNA (Additional file 2). The set B allelic dropout rate across loci was 0.16 and the false allele rate across loci was 0.043. The genotype error rates for both set A and set B were within the range of 48 h depredation rates as documented by [34]. We successfully genotyped three of the six nests (50 %) and three of the three carcasses (100 %), and each genotype had no fewer than two missing loci out of ten (Table 2). Coyotes which were removed and for which genotypes were obtained were on average within 18.56 km (range 0–56.82 km) of the nearest depredated nest or carcass at time of removal. Coyotes in Wyoming have large annual home ranges (13.12 ± 1.59 km^2^, [35]), therefore our expectation to match removed individuals with depredated nests is in agreement with coyote biology. These were compared to 27 tissue genotypes and 28 fecal genotypes from Orning [20]. We were unable to match any genotypes from eggs and carcasses to captured coyotes or fecal samples collected in the study area. None of the nest or carcass predator genotypes matched each other.

## Discussion

The goal of this study was to test the concept that non-invasive genetic sampling can be used as a forensic tool to identify predators of ground-nesting birds. We employed both mitochondrial and nuclear DNA to assist field identification of sage-grouse nest predators, and we identified mammalian predators in 79 % of depredation events. When we compared the results of the genetic analyses to species identifications made in the field from physical evidence and camera data, the molecular results agreed with identifications made from physical evidence in four cases, all identified as coyote, and also identified the nest predators in four cases where species identification from physical evidence was deemed indeterminate (Table 2). We determined that five mammalian species had contact with 11 depredated nests (Table 2). In the majority of the cases the species was a canid, but we encountered two events, one nest and one carcass, where we were unable to resolve the canid species identification (e.g., wolf or coyote). Both cases were most likely coyote because wolves have not been documented in the study area [33]. We could not distinguish the canid species because some mtDNA haplotypes found in wolves in eastern North America and coyotes are nearly identical, which is thought to be a result of historic hybridization or incomplete lineage sorting [36].

The molecular method we employed was successful in supplementing field and camera identifications of nest predators by either confirming or providing identification when other methods proved inconclusive. There were only two disagreements between physical evidence and our results, in one case the field identification suggested bird or bobcat (*Lynx rufus*) as the predator but we identified cow, and in the second case the field identification was cow but we identified coyote. Camera data provided evidence for predator identification in eight out of 14 nests and was not applied to carcasses (Table 2). The camera data and the molecular identifications unequivocally agreed in four of the cases and all of these were coyote. In one case the camera captured a close-up of the face of a potential predator but from the photo we could not differentiate whether it was a striped skunk or an American badger (*Taxidea taxus*). In this case the molecular data clarified the camera data by determining that it was a striped skunk. There was a disagreement in only one case where the molecular data identified domestic dog DNA but the camera captured a photo of a weasel (*Mustela* sp.) near the nest. Possible reasons for disagreements between datasets are likely due to difficulties with predator species identification in the field from nest remains, non-mammalian predators, camera failure, or DNA isolated from a scavenger instead of the nest predator. Therefore, we recommend combining molecular data and cameras to increase the success in identifying mammalian predators of ground-nesting birds, similar to the approach by Steffens et al. [13] but applying a mulit-locus method to improve individual and species identification success. Combining these two techniques provides valuable insight for management decisions to facilitate protection of threatened and endangered species.

All of the species we detected have been previously documented as nest predators [37, 38]. The literature contains multiple reports of both coyotes and striped skunks feeding upon eggs of ground-nesting birds [39, 40]. The high rate of coyote depredation detected in this study was not surprising given that this species is known to be abundant in the study areas and they have been documented eating sage-grouse eggs [8, 20]. Even though dogs are not considered a common predator of sage-grouse nests [8, 37, Orning and Young, in review], we detected DNA evidence of domestic dogs from three depredated nests. Domestic dogs have been documented disturbing nests of both ground-nesting birds and sea turtles [41, 42], but not sage-grouse eggs. These nests were not close to human dwellings, the area is 30–40 miles from a populated area, but human activities that included the presence of dogs, such as oil and gas development, recreation, and livestock, were regularly observed in the area of the nesting sage-grouse (E. O. Orning; personal observation). However, the small sample size of this study may have artificially amplified the apparent impacts that dogs have on sage-grouse nests. Thus the dog results obtained herein should be interpreted with caution when considering management strategies. One approach that could easily reduce the potential for human-caused losses of this nature would be to increase public awareness of sage-grouse nesting by limiting human and pet access to areas during critical nesting periods. Deer mice, in particular native *Peromyscus* spp., have been recognized as preying upon ground-nesting sea bird eggs but they also visit depredated nests as secondary consumers [8, 43]. We also documented cow DNA from one nest. Cattle are possible ground-nesting bird egg predators [44]. However, cattle activity and feces were in close proximity to some of the nests and Orning [20] collected video of cattle investigating sage-grouse nests so whether this event was predation or contamination is unclear. Finally, we detected human DNA from a few of the nests. The sensitivity of the general mammalian primers used for this study must be taken into account when conducting a molecular forensic study. Wildlife genetics laboratories should also apply the same stringent protocols of sample collection and processing required in the human forensics field [45]. Further, approaches can be applied in the laboratory to prevent amplification of human DNA such as species-specific PCR primers [5], human-blocking PCR primers [46], and metabarcoding with high-throughput sequencing technology.

There are multiple reasons that might explain why our molecular technique was not 100 % successful in identifying nest predators. The most likely explanation is that the DNA had degraded beyond our detection ability. If the eggs were collected too long after the depredation event, and depending on weather conditions, the DNA could degrade quickly [47]. Increasing the number of visitations to nests to shorten the time intervals between predation and discovery is unlikely to be a feasible approach to limit DNA degradation. Intensified human disturbance can increase the chances of nest abandonment or predation which would be counterproductive to conservation goals [7, 48]. One way to increase the success rate of detecting degraded DNA would be to target a smaller fragment. However, this approach has drawbacks because resolution can be lost for differentiating recently derived species when using shorter fragments of DNA but this shortcoming could be addressed by using genes with higher mutation rates. Increasing the sensitivity of the PCR assay through optimization is one way to account for DNA degradation. For example, we increased the number of PCR cycles to 52 for microsatellite Set B which allowed us to obtain a higher percentage of full genotypes. However this high number of cycles could increase the chance of false alleles. In fact, any optimization strategy could increase non-specific amplification which could decrease the accuracy of species identification.

Another explanation for failure to detect the predator species might be that the nest predator was avian. The primers we used were mammal-specific, thus we were not able to amplify avian DNA. We did have one case where field reports suggested the nest predator was a raven (*Corvus corvax*) or a snake and another where the camera identified a raven around the nest, but could we did not obtain molecular species identification from either nest. Using this method to determine if the predator was a reptile (i.e., snake) or corvid may not be practical as both taxa are known to consume the entire egg on site or carry it off thus there is a possibility that no fragments will be left behind to swab [49, 50].

We foresee multiple continuations of this study to increase the thoroughness and robustness of predator species identification. The first and most obvious is to increase the sample size. We acknowledge that the sample size in this study is quite small, but our goal was to prove the concept rather than thoroughly quantify the diversity of predators on sage-grouse eggs and adults. To rigorously estimate nest predation rates, and provide a control for identifying predators of sage-grouse adults, which was lacking from this study, there would need to be a larger study with more cameras placed around leks and on more leks throughout the range of the species. Another follow-up study would be to test DNA degradation rates by having captive predators deposit saliva on eggshells and carcasses. This would allow one to establish a reasonable time since depredation and evaluate the accuracy of predator species identification from adult carcasses as these are usually opportunistic samples and filming these depredation events is unlikely. To increase the breadth of predator taxa identified from nest remains and carcasses one could apply primers that amplify avian DNA. Avian predator DNA has been successfully sampled from black-fronted tern (*Chlidonias albostriatus*) eggshells [13], so this approach may also be feasible for sage-grouse. The challenge of this approach is that DNA from the prey species could also be amplified which would obscure predator identification as seen in Steffens et al. [13]. Thus employing nesting species blocking primers may be an approach to decrease the chances of avian predator and prey co-amplification [46].

## Conclusions

The ultimate goals of conservation plans are to halt decline and facilitate recovery of species at risk. Before any management strategy can be effective, one must identify the proximate threats. In many cases, the ecosystems within which the threated species exist are out of balance, usually due to human activities. This could then lead to higher impacts from some community interactions such as predation [51]. The predator identification method identified in our study could help sage-grouse management by understanding if predation, and in particular certain predator species, have inordinate impacts on sage-grouse recruitment. If determined to be a major factor then implementation of a predator management program could help the recovery of the sage-grouse. In many cases knowledge of predation alone is insufficient to inform conservation [52].Predation may not be the reason for decline, but a management strategy like temporary predator control may aid nest success until the habitat is restored to the ability to naturally support sage-grouse. This study and Orning [20] were designed to understand predation within the Wyoming management units for sage-grouse and one option with these units is predator control. Orning [20] found that coyote control did not have large impacts on survival of hens or nests. However, this might not be true in other parts of the species range as it encompasses a large portion of the western United States. Including a tool such as molecular identification of predators can help managers then choose if predator control is the right strategy in their areas as predation may not be the actual limiting factor to population growth [52].

Predator management may be necessary in human-altered landscapes where ecosystems and coevolutionary processes are out of balance [53]. However, indirect effects of predator management such as apparent competition [50], exploitative competition [54], mesopredator release [55–57], and disruption of social structure could increase predation [58], thus, care must be taken when implementing such strategies as conservation tools. Identifying the predators that are negatively impacting a species of concern is a critical step in understanding the larger ecological questions about predator–prey dynamics, the impacts of predation, and the effects of management strategies (like predator removal) on recruitment. Nest and adult predator identification is a challenging endeavor considering the lack of species-specific signs at nests and the difficulty differentiating predators from scavengers using DNA evidence. The results of this study suggest that the best approach is to utilize multiple lines of evidence consisting of field surveys, camera monitoring of depredation events, and DNA forensics-based methods such as the one described here. The molecular method we tested has some advantages over field-based methods in that environmental disturbance is minimized and improved ease of collection as animals do not need to be handled. The combination of field and laboratory approaches will hopefully increase success of nest predator identification within a target study area and may be applied to management of any ground-nesting bird species.

## Availability of supporting data

DNA sequences were deposited in NCBI GenBank under accession numbers KT946968-KT946984. Microsatellite genotypes are submitted as supplemental information.

## Additional files

10.1186/s13104-015-1797-1 Predator genotypes in genepop format with each PCR replicate included for microsatellites set A in genepop format (.prn).
10.1186/s13104-015-1797-1 Predator genotypes in genepop format with each PCR replicate included for microsatellites set B in genepop format (.prn).

## References

[1] Taberlet P, Waits LP, Luikart G (1999). Noninvasive genetic sampling: look before you leap. Trends Ecol Evol.

[2] Beja-Pereira A, Oliveira R, Alves PC, Schwartz MK, Luikart G (2009). Advancing ecological understandings through technological transformations in noninvasive genetics. Mol Ecol Resour.

[3] Blejwas KM, Williams CL, Shin GT, McCullough DR, Jaeger MM. Salivary DNA evidence convicts breeding male coyotes of killing sheep. J Wildlife Manage. 2006;70(4):1087–93. doi:10.2193/0022-541X(2006)70[1087:SDECBM]2.0.CO;2.

[4] Onorato D, White C, Zager P, Waits LP. Detection of predator presence at elk mortality sites using mtDNA analysis of hair and scat samples. Wildl Soc Bull. 2006;34(3):815–20. doi:10.2193/0091-7648(2006)34[815:DOPPAE]2.0.CO;2.

[5] Mumma MA, Soulliere CE, Mahoney SP, Waits LP (2014). Enhanced understanding of predator–prey relationships using molecular methods to identify predator species, individual and sex. Mol Ecol Resour.

[6] Imazato H, Onuma M, Nagamine T, Nakaya Y (2012). Molecular species identification of predators of endangered species on Okinawa-Jima island. Mammal Study.

[7] Larivière S (1999). Reasons why predators cannot be inferred from nest remains. The Condor.

[8] Coates PS, Connelly JW, Delehanty DJ (2008). Predators of greater sage-grouse nests identified by video monitoring. J Field Ornithol.

[9] Boulton RL, Cassey P (2006). An inexpensive method for identifying predators of passerine nests using tethered artificial eggs. New Zealand J Ecol.

[10] Sanders MD, Maloney RF (2002). Causes of mortality at nests of ground-nesting birds in the Upper Waitaki Basin, South Island, New Zealand: a 5-year video study. Biol Conserv.

[11] Séquin ES, Jaeger MM, Brussard PF, Barrett RH (2003). Wariness of coyotes to camera traps relative to social status and territory boundaries. Can J Zool.

[12] Bush K, Vinsky M, Aldridge C, Paszkowski C (2005). A comparison of sample types varying in invasiveness for use in DNA sex determination in an endangered population of greater Sage-Grouse (*Centrocercus uropihasianus*). Conserv Genet.

[13] Steffens KE, Sanders MD, Gleeson DM, Pullen KM, Stowe CJ (2012). Identification of predators at black-fronted tern *Chlidonias albostriatus* nests, using mtDNA analysis and digital video recorders. New Zealand J Ecol.

[14] Garton EO, Connelly JW, Horne JS, Hagen CA, Moser A, Schroeder MA, Knick ST, Connelly JW (2011). Greater sage-grouse population dynamics and probablity of persistence. Studies in avian biology.

[15] Schroeder MA, Aldridge CL, Apa AD, Bohne JR, Braun CE, Bunnell SD (2004). Distribution of sage-grouse in North America. Condor.

[16] USFWS. 12-month finding for petitions to list the greater sage-grouse (*Centrocercus urophasianus*) as threatened or endangered. 2010. p. 13909–4014.

[17] Taylor RL, Walker BL, Naugle DE, Mills LS (2012). Managing multiple vital rates to maximize greater sage-grouse population growth. J Wildl Manag.

[18] Moynahan BJ, Lindberg MS, Rotella JJ, Thomas JW (2007). Factors affecting nest survival of greater sage-grouse in Northcentral Montana. J Wildl Manag.

[19] Beck JL, Reese KP, Connelly JW, Lucia MB. Movements and survival of juvenile greater sage-grouse in southeastern Idaho. Wildlife Society Bulletin (1973–2006). 2006;34(4):1070–8. doi:10.2307/4134318.

[20] Orning EK (2013). Effect of predator removal on greater sage-grouse (*Centrocercus urophasianus*) ecology in the Bighorn Basin conservation area of Wyoming.

[21] Smith MF, Patton JL (1993). The diversification of South American murid rodents: evidence from mitochondrial DNA sequence data for the akodontine tribe. Biol J Linn Soc.

[22] Vilà C, Amorim IR, Leonard JA, Posada D, Castroviejo J, Petrucci-Fonseca F (1999). Mitochondrial DNA phylogeography and population history of the grey wolf *Canis lupus*. Mol Ecol.

[23] Townzen JS, Brower AVZ, Judd DD (2008). Identification of mosquito bloodmeals using mitochondrial cytochrome oxidase subunit I and cytochrome b gene sequences. Med Vet Entomol.

[24] García-Moreno J, Matocq MD, Roy MS, Geffen E, Wayne RK (1996). Relationships and genetic purity of the endangered Mexican wolf based on analysis of microsatellite loci. Conserv Biol.

[25] Francisco LV, Langsten AA, Mellersh CS, Neal CL, Ostrander EA (1996). A class of highly polymorphic tetranucleotide repeats for canine genetic mapping. Mamm Genome.

[26] Brownstein MJ, Carpten JD, Smith JR (1996). Modulation of non-templated nucleotide addition by Taq DNA polymerase: primer modifications that facilitate genotyping. Biotechniques.

[27] Taberlet P, Griffin S, Goossens B, Questiau S, Manceau V, Escaravage N (1996). Reliable genotyping of samples with very low DNA quantities using PCR. Nucleic Acids Res.

[28] Navidi W, Arnheim N, Waterman MS (1992). A multiple-tubes approach for accurate genotyping of very small DNA samples by using PCR: statistical considerations. Am J Hum Genet.

[29] Valière N (2002). GIMLET: a computer program for analysing genetic individual identification data. Mol Ecol Notes.

[30] Falush D, Stephens M, Pritchard JK (2003). Inference of population structure using multilocus genotype data: linked loci and correlated allele frequencies. Genetics.

[31] Pritchard JK, Stephens M, Donnelly P (2000). Inference of population structure using multilocus genotype data. Genetics.

[32] Peakall R, Smouse PE (2012). GenAlEx 6.5: genetic analysis in Excel. Population genetic software for teaching and research—an update. Bioinformatics.

[33] Wyoming Game and Fish Department, National Park Service, USDA-APHIS-Wildlife Services, U.S. Fish and Wildlife Service. 2014 Wyoming gray wolf population monitoring and management interim report. Cheyenne, Wyoming, USA: Wyoming Game and Fish Department, 2015.

[34] Harms V, Nowak C, Carl S, Muñoz-Fuentes V (2015). Experimental evaluation of genetic predator identification from saliva traces on wildlife kills. J Mammal.

[35] Berger KM, Gese EM (2007). Does interference competition with wolves limit the distribution and abundance of coyotes?. J Anim Ecol.

[36] Fain S, Straughan D, Taylor B. Genetic outcomes of wolf recovery in the western Great Lakes states. Conserv Genet. 2010;11(5):984–90.

[37] Hagen C (2011). Predation on greater sage-grouse: facts, process, and effects. Stud Avian Biol.

[38] Schroeder MA, Baydack RK (2001). Predation and the management of prairie grouse. Wildl Soc Bull.

[39] Pasitschniak-Arts M, Messier F (1995). Predator identification at simulated waterfowl nests using inconspicuous hair catchers and wax-filled eggs. Can J Zool.

[40] Johnson DH, Sargeant AB, Greenwood RJ (1989). Importance of individual species of predators on nesting success of ducks in the Canadian Prairie Pothole Region. Can J Zool.

[41] Yanes M, Suárez F (1996). Incidental nest predation and lark conservation in an Iberian semiarid shrubsteppe. Conserv Biol.

[42] Fowler LE. Hatching success and nest predation in the green sea turtle, *Chelonia mydas*, at Tortuguero, Costa Rica. Ecology. 1979;946–55.

[43] Blight LK, Ryder JL, Bertram DF (1999). Predation on rhinoceros auklet eggs by a native population of *Peromyscus*. The Condor..

[44] Nack JL, Ribic CA (2005). Apparent predation by cattle at grassland bird nests. Wilson Bull.

[45] Ogden R, Dawnay N, McEwing R (2009). Wildlife DNA forensics—bridging the gap between conservation genetics and law enforcement. Endangered Species Res.

[46] Vestheim H, Jarman SN (2008). Blocking primers to enhance PCR amplification of rare sequences in mixed samples–a case study on prey DNA in Antarctic krill stomachs. Front Zool.

[47] Lindahl T (1993). Instability and decay of the primary structure of DNA. Nature.

[48] Major RE, Kendal CE (1996). The contribution of artificial nest experiments to understanding avian reproductive success: a review of methods and conclusions. Ibis.

[49] Montevecchi WA (1976). Egg size and the egg predatory behaviour of crows. Behaviour.

[50] Staller EL, Palmer WE, Carroll JP, Thornton RP, Sisson DC (2005). Identifying predators at northern bobwhite nests. J Wildl Manag.

[51] Berlow EL (1999). Strong effects of weak interactions in ecological communities. Nature.

[52] Vucetich JA, Smith DW, Stahler DR (2005). Influence of harvest, climate and wolf predation on Yellowstone elk, 1961–2004. Oikos.

[53] Reynolds JC, Tapper SC (1996). Control of mammalian predators in game management and conservation. Mammal Rev.

[54] MacArthur R, Levins R. The limiting similarity, convergence, and divergence of coexisting species. Am Natural. 1967; 377–85.

[55] Mezquida ET, Slater SJ, Benkman CW. Sage-grouse and indirect interactions: potential implications of coyote control on sage-grouse populations. Condor. 2006;108(4):747–59. doi:10.1650/0010-5422%282006%29108%5B747:SAIIPI%5D2.0.CO;2.

[56] Prugh LR, Stoner CJ, Epps CW, Bean WT, Ripple WJ, Laliberte AS et al. The rise of the mesopredator. BioScience. 2009;59(9):779–91. doi:10.1525/bio.2009.59.9.9.

[57] Conner LM, Rutledge JC, Smith LL. Effects of Mesopredators on Nest Survival of Shrub-Nesting Songbirds. J Wildl Manag. 2010;74(1):73–80. doi:10.2193/2008-406.

[58] Mitchell BR, Jaeger MM, Barrett RH. Coyote depredation management: current methods and research Needs. Wildlife Soc Bull (1973–2006). 2004;32(4):1209–18. doi:10.2307/3784759.

